# simmrd: An open-source tool to perform simulations in Mendelian randomization

**DOI:** 10.1002/gepi.22544

**Published:** 2024-01-23

**Authors:** Noah Lorincz-Comi, Yihe Yang, Xiaofeng Zhu

**Affiliations:** Department of Population and Quantitative Health Sciences, Case Western Reserve University, Cleveland, Ohio, USA

**Keywords:** bias, causal inference, instrumental variables, Mendelian randomization, simulation

## Abstract

Mendelian randomization (MR) has become a popular tool for inferring causality of risk factors on disease. There are currently over 45 different methods available to perform MR, reflecting this extremely active research area. It would be desirable to have a standard simulation environment to objectively evaluate the existing and future methods. We present simmrd, an open-source software for performing simulations to evaluate the performance of MR methods in a range of scenarios encountered in practice. Researchers can directly modify the simmrd source code so that the research community may arrive at a widely accepted framework for researchers to evaluate the performance of different MR methods.

## 1 | INTRODUCTION

Mendelian randomization (MR) is a genetic instrumental variable method that has become a popular statistical tool for estimating causal effects of risk factors on disease (Richmond & Davey Smith, 2022; Sanderson, Glymour, et al., 2022). The validity of inferences made using MR relies heavily on the satisfaction of three primary assumptions (de Leeuw et al., 2022): the genetic instruments are (i) strongly associated with the exposure(s), (ii) not associated with the outcome conditional on the exposures, and (iii) not associated with any confounders of the exposure(s)-outcome relationship(s) (Davies et al., 2015; Hemani et al., 2018; Morrison et al., 2020; Sanderson et al., 2021). Violating any of these assumptions can lead to bias in causal estimation, which may inflate false positive and/or false negative rates (Bowden et al., 2015). Three additional challenges are present in MR analyses: (a) some individuals may have been present in both the exposure(s) and outcome Genome-Wide Association Study (GWAS) which leads to sample overlap bias (Burgess, Davies, et al., 2016), (b) applying strict instrumental variable (IV) selection criteria to satisfy assumption (i) above can introduce a “winner’s curse” bias (Jiang, Gill, et al., 2022), and (c) strong linkage disequilibrium (LD) between IVs (Gkatzionis et al., 2023), and its imprecise estimation using reference panels, can inflate false positive rates (Gleason et al., 2021).

At least 45 statistical methods for performing MR have been introduced in the literature to address different subsets of assumptions (i)–(iii) and scenarios (a)–(c) in either the single-exposure (univariable) or multiple-exposure (multivariable) settings (Boehm & Zhou, 2022). Clearly, the literature is saturated with a variety of MR estimators. In each of the initial studies introducing these methods, simulation was performed to evaluate the performance of new and existing methods. The variability of simulation settings reported in the literature is vast. Some use 50 or fewer single-nucleotide polymorphisms (SNPs) to explain most of phenoptypic (Verbanck et al., 2018; Zhu et al., 2021) or genetic (Lin et al., 2023) variance while others have used hundreds (Lorincz-Comi et al., 2023) or even thousands (Mounier & Kutalik, 2023) of SNPs. Additionally, some have used GWAS sample sizes no larger than 500 (Deng et al., 2022) while others have used GWAS sample sizes no less than 100k (Xu et al., 2021). For example, some have 50 or less IVs in simulation (Lin et al., 2023; Sanderson et al., 2019; Sanderson, Richardson, et al., 2022), but experience with real data analyses with multiple exposures suggests that hundreds or even thousands of SNPs meeting the valid IV conditions may be identified (Davies et al., 2019; Lorincz-Comi et al., 2023). Since weak IV bias generally becomes more severe as more IVs are used (Davies et al., 2015; Lorincz-Comi et al., 2023), multivariable Mendelian randomization (MVMR) simulations in which very few IVs are used may unrealistically reflect optimal performance of MVMR methods which may break down in practice.

Some of these issues arise from researchers reproducing the marginally realistic simulation settings of others as described above (e.g., Zhu et al., 2021, reproduced Verbanck et al., 2018; Lin et al., 2023, reproduced Grant & Burgess, 2021). Other challenges may exist because of a unified framework for performing simulations in MR. Both explanations have produced a literature of results potentially sensitive to the unique simulation conditions that may or may not mirror reality. Indeed, some independent simulations intended to mimic the same reality with nearly identical reported simulation settings have even produced conflicting results. As example, the literature contains some conflicting evidence about the bias in dIVW in the absence of sample overlap and horizontal pleiotropy (Mounier & Kutalik, 2023; Ye et al., 2021); the power of MR-Robust in the presence of 50% invalid IVs, being either less than 20% or greater than 80% (Lin et al., 2023; Slob & Burgess, 2020); or MR-RAPS having Type I error greater than 80% or controlled at 5% in the presence of horizontal pleiotropy (Qi & Chatterjee, 2021; Xue et al., 2021).

We introduce simmrd, an open-source and flexible tool for generating data to use in MR simulations. The last time a tool such as this was introduced was MR_predictor 10 years ago (Voight, 2014), however, MR_predictor cannot accommodate horizontal pleiotropy, sample overlap, multiple exposures, weak instruments, correlated IVs, imprecise LD estimation, or winner’s curse. simmrd can accommodate each of these and even more scenarios simultaneously in either the univariable Mendelian randomization (UVMR) or MVMR settings. With no modern standard set in the literature, the purpose of the open-source simmrd software is to begin the construction of a standard simulation environment in which fair comparisons can be made between competing MR estimators in different settings that are encountered in practice.

## 2 | SIMULATION MODELS

**FIGURE 1:**
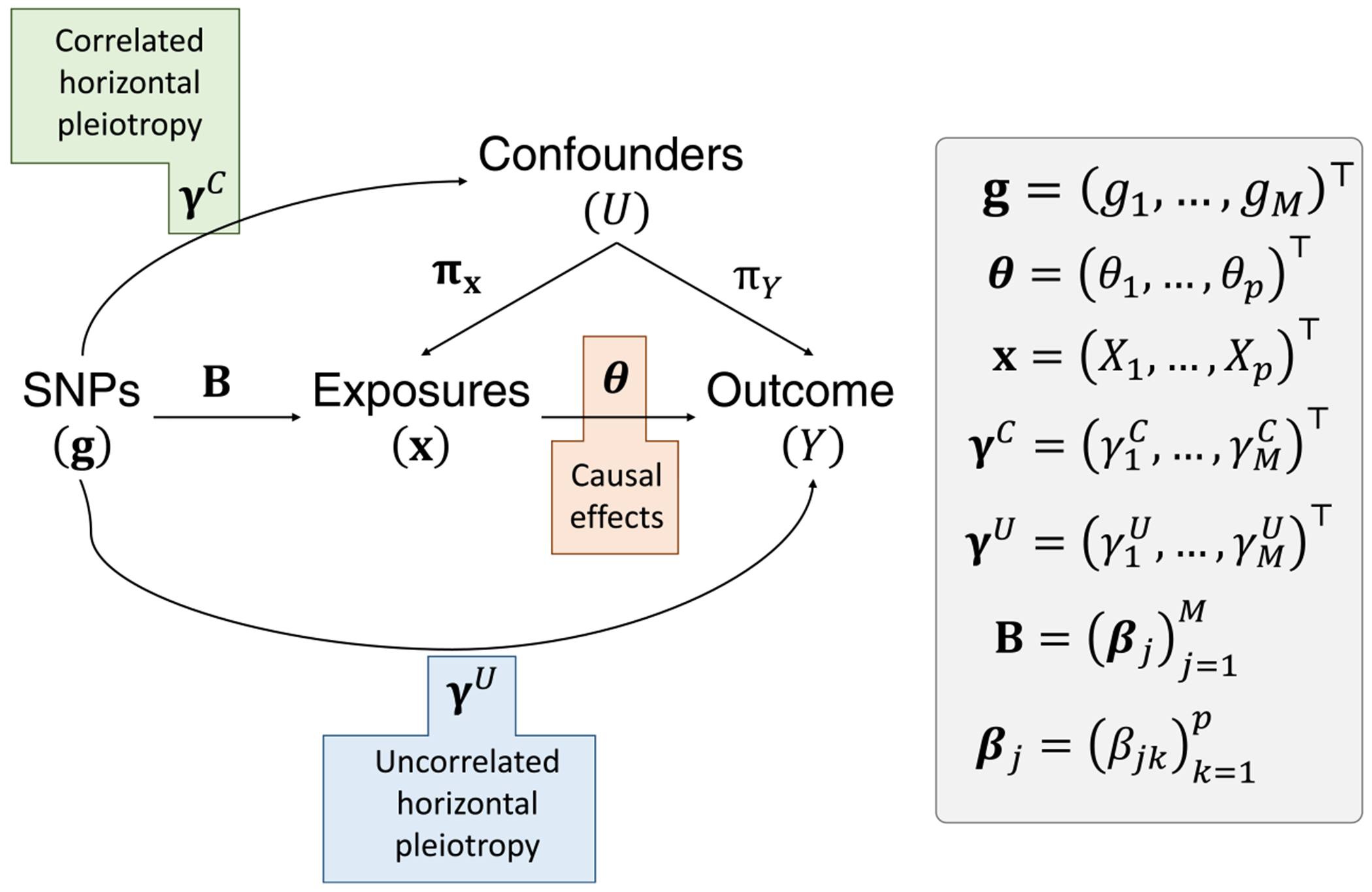
This DAG is used to produce the simulation models described in Section 2. Users of simmrd can modify all parameters that are present in this DAG using the generate_individual() function, or most using the generate_summary() function. The parameter B represents true genetic associations between the SNP genotypes in g and each of the exposures in x, and θ represents the true causal effects of the exposures on the outcome Y conditional on confounding U, whose effects on the exposures and outcome are respectively $\pi_{x}$ and $\pi_{Y}$. The parameter $\gamma^{C}$ represents correlated horizontal pleiotropy and $\gamma^{U}$ represents uncorrelated horizontal pleiotropy.

The simulation data-generating process is based on the directed acyclic graph (DAG) in Figure 1. This DAG can be represented by the following structural models:

(1) $$U=g^{⊤}\gamma^{C}+\epsilon_{U},$$

(2) $$x=B^{⊤}g+\pi_{x}U+\epsilon_{x},$$

(3) $$Y=x^{⊤}\theta +\pi_{Y}U+g^{⊤}\gamma^{U}+\epsilon_{Y}$$

(4) $$=\alpha^{⊤}g+{\bar{\epsilon }}_{Y},$$

where U is a confounder of the relationship between exposure(s) x and outcome Y,θ represents the corresponding causal effect(s) of the exposure(s) on the outcome, B represents true associations between g and x, and $\gamma^{C}$ and $\gamma^{U}$ are respectively correlated horizontal pleiotropy (CHP) and uncorrelated horizontal pleiotropy (UHP) effects. The aim of MR is to estimate θ using GWAS summary data corresponding to B and α, which at minimum includes association estimates and their corresponding standard error estimates.

**FIGURE 2:**
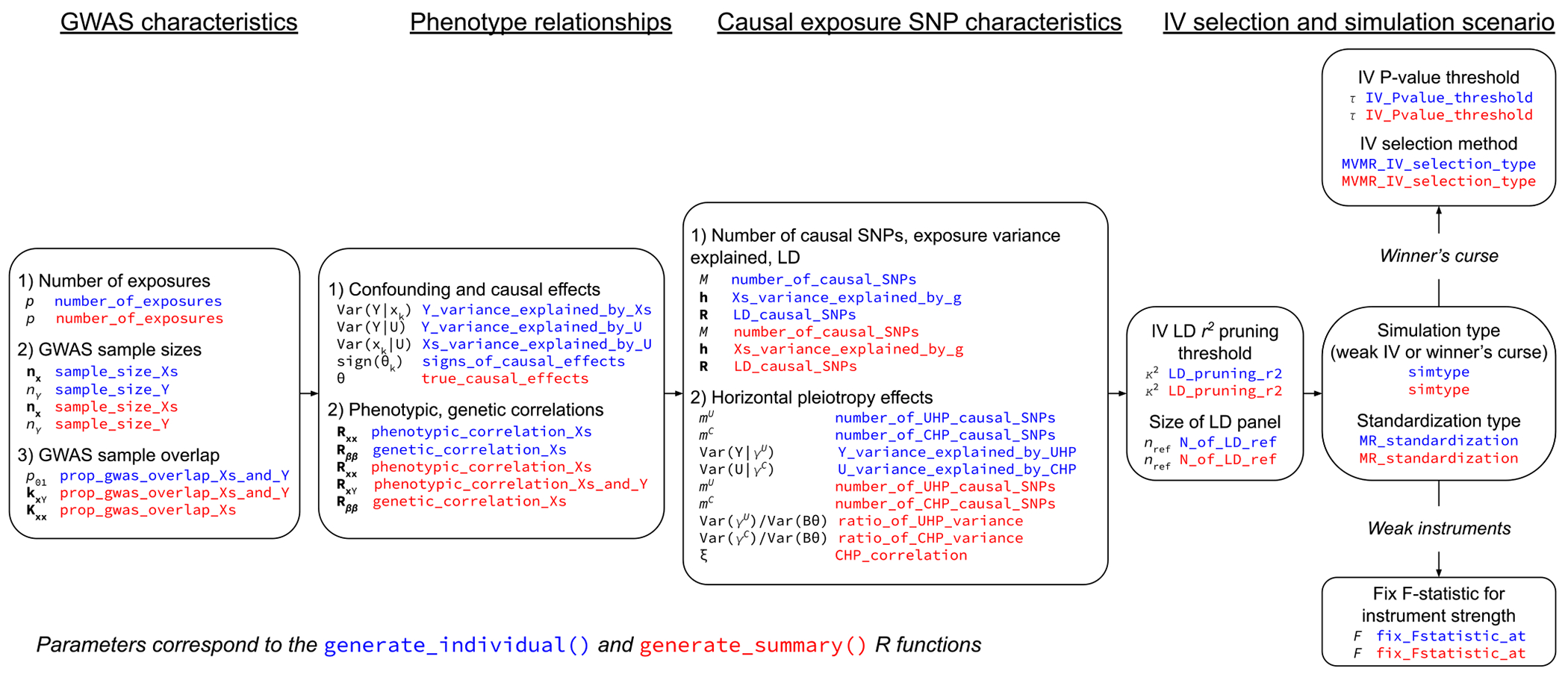
This figure provides a conceptual overview of the simmrd data-generating process and the parameters to set when using the generate_individual() (in blue) or generate_summary() functions (in red). Brief descriptions of how each parameter is set and used is provided in the appendix and complete examples of how to generate simulated data using generate_summary() and generate_individual() are available at https://github.com/noahlorinczcomi/simmrd.

GWAS summary data can be generated either by drawing individual-level data from Equations (1) to (4) above then performing GWAS on them, or by directly generating the GWAS summary statistics from fully specified distributions which place some assumptions on the models in Equations (1)–(4). Since in some cases users of simmrd may want the fully flexibility of the models above, but in others only require a simplified data-generating process which is more computationally efficient, we provide users with both options. Users can indirectly generate GWAS summary data from individual-level data using the generate_individual() function, or directly generate GWAS summary data using the generate_summary() function. Algorithm 1 shows the complete data-generating process that generate_individual() uses and Algorithm 2 shows the data-generating process that generate_summary() uses. Section 5 provides more computational details and outlines the steps required to use each function. The parameters that users can set in the generate_individual() and generate_summary() functions is presented in Figure 2. This figure demonstrates that generate_individual() allows users with greater flexibility in specifying the exact relationships of the confounder with the exposures and outcome compared to generate_summary(). These two approaches also differ in their setting of the true causal effect parameters. Using generate_individual(), users specify pairwise conditional variances to parameterize the models in Equations (1)–(4). Model parameters are then exactly determined by assuming the exposures, confounder, and outcome each have variance 1. Using generate_summary(), users can directly specify the true causal effect sizes. Additionally, for computational simplicity, all exposures GWAS samples generated using generate_individual() completely overlap, whereas generate_summary() can accommodate an arbitrary overlap structure between all exposures and the outcome.

**Algorithm 1:** Pseudo-code of simulated GWAS summary-level data using the generate_individual() function.

**Require**: Cov(x∣U), exposure SNP heritabilties $h={\left(h_{1}^{2},\ldots ,h_{p}^{2}\right)}^{⊤},Var\left([Y|U]|x_{k}\right)$ for each k=1,…,p exposures, $sign\left(\theta_{k}\right),p\times p$ matrices of phenotypic and genetic correlation between the exposures $R_{xx}$ and $R_{\beta \beta }$, respective exposure(s) and outcome GWAS sample sizes $n_{1}$ and $n_{0}$ and their proportion of overlap $p_{01}$, the number of causal exposure SNPs M and their LD structure R, the number of individuals in the LD reference panel $n_{ref}$, the number of UHP and CHP causal exposure SNPs $m^{U}$ and $m^{C}$, the variance in Y and U respectively explained by UHP and CHP SNPs $\sigma_{\gamma^{U}}^{2}$ and $\sigma_{\gamma^{C}}^{2}$, LD pruning threshold for IV selection $\kappa^{2}$, the simulation type (either winner’s curse or weak IVs), the type of standardization to apply to the MR summary data (*Z*-score standardization or no standardization), the *p*-value threshold for IV selection if performing winner’s curse simulations τ, the F-statistic for IV strength of association with exposures (F) if performing a weak instrument simulation.
**Preliminaries**: All phenotypes ($x^{⊤},Y,U,g^{⊤}$) will have mean 0 and variance 1. UHP, CHP, and SNPs that are otherwise valid IV candidates are put into mutually exclusive groups (see Figure 2).
**Step 1.** Draw $n_{0}+n_{1}$ genotypes from $g_{j}∼\mathrm{Binomial}(2,0.3)$ and standardize to $E\left(g_{j}\right)=0,Var\left(g_{j}\right)=1;j=1,\ldots ,M$
**Step 2.** Draw CHP effects for $m^{C}$ SNPs from $\gamma_{j}^{C}∼\mathrm{Uniform}(-1,1)$ and re-scale to explain $\sigma_{\gamma^{C}}^{2}$ variance in U
**Step 3.** Draw UHP effects for $m^{U}$ SNPs from $\gamma_{j}^{U}∼\text{Uniform}(-1,1)$ and re-scale to explain $\sigma_{\gamma^{U}}^{2}$ variance in Y
**Step 4.** Draw SNP associations with the exposures (**B**) from a matrix normal distribution with row-wise covariance R and column-wise covariance $M^{-1}{DR}_{\beta \beta }D$, where $D=diag\left(h_{1},\ldots ,h_{p}\right)$, and re-scale the columns such that ${\left[diag\left(B^{⊤}B\right)\right]}_{k}=h_{k}^{2}$
**Step 5.** Set $\pi^{Y}=-1$ and $\theta_{k}=sign\left(Cov\left[Y,x_{k}\right]\right)$ and re-scale each to achieve $Var\left([Y|U]|x_{k}\right)$ for k=1,…,p
**Step 6.** Define sets ${𝒫}_{x}$ and ${𝒫}_{Y}$ of simulated individuals to respectively use in the exposure and outcome GWAS, of which $p_{01}\times min\left(n_{0},n_{1}\right)$ elements will overlap
**Step 7.** Perform GWAS using OLS on phenotypes $Y_{{𝒫}_{Y}}$ and $x_{{𝒫}_{x}}$
**Step 8.** Standardize all GWAS summary data according to user input (one of “z” or “none”)
**Step 9.** Perform IV selection according to LD threshold $\kappa^{2}$, *p*-value thresohld τ, and *F*-statistic *F* to obtain IV set 𝒮
**Step 10.** Draw the estimated LD matrix for set of IVs from $Wishart\left(n_{\text{ref}},R_{𝒮}\right)$ and standardize to be the correlation matrix ${\hat{R}}_{𝒮}$
**Ensure**: The set of IVs 𝒮; their standardized GWAS estimates for the exposures (${\hat{B}}_{𝒮}$) and the outcome $\left({\hat{\alpha }}_{𝒮}\right)$ as the R objects bx and by; the corresponding GWAS-estimated standard errors $\hat{SE}\left({\hat{B}}_{𝒮}\right)$ and $\hat{SE}\left({\hat{\alpha }}_{𝒮}\right)$ as bxse and byse; indications for each IV if it was generated as a valid, UHP, or CHP SNP in IVType; true causal effects θ as theta; the matrix of correlations between GWAS estimation errors Ω as RhoME, with elements corresponding to the outcome in the first row and column; the matrices of true and estimated LD between the IVs $R_{𝒮}$ and ${\hat{R}}_{𝒮}$ as LDMatrix and LDhatMatrix.

**Algorithm 2:** Pseudo-code of simulated GWAS summary-level data using the generate_summary() function.

**Require**: The number of exposures (p), GWAS sample sizes for all exposures ($\left(n_{x}={\left[n_{X1},\ldots ,x_{Xp}\right]}^{⊤}\right)$) and the outcome $\left(n_{Y}\right)$ and their proportions of overlapping participants $\left(k_{xY}={\left[k_{1},\ldots ,k_{p}\right]}^{⊤}\right)$, the p×p matrix of overlap proportions between exposure GWASs $\left(K_{xx}\right)$, the matrix of phenotypic correlations between the outcome and all exposures ($R_{xY}$), SNP heritability of the exposures $h=\left(h_{1}^{2},\ldots ,h_{p}^{2}\right)$, the matrix of genetic correlation between the exposures $\left(R_{\beta \beta }\right)$, the number of SNPs causally associated with the exposures (M), the number of causal SNPs which have UHP $\left(m^{U}\right)$ and CHP $\left(m^{C}\right)$ effects, the proportion of total MR variance (see **Preliminaries** for a definition) due to UHP $\left(\rho^{U}\right)$ and CHP $\left(\rho^{C}\right)$, the correlation between CHP and non-CHP effects $\left(\zeta^{C}\right)$, the p-length vector of true causal effects (θ), LD pruning threshold for IV selection $\left(\kappa^{2}\right)$, simulation type (either winner’s curse or weak IVs), type of standardization to apply to the MR summary data (Z-score standardization or no standardization), p-value threshold for IV selection if performing winner’s curse simulations (τ), F-statistic for IV strength of association with exposures (F) if performing a weak instrument simulation, the LD structure (R) between the M causal SNPs and the number of independent LD blocks (s), and the sample size of the LD reference panel $\left(n_{\text{ref}}\right)$.
**Preliminaries**: Data is generated under the assumption that all phenotypes ($x^{⊤},Y,U,g^{⊤}$) have mean 0 and variance 1. UHP, CHP, and SNPs that are otherwise valid IV candidates are put into mutually exclusive groups (see Figure 2). Where the true MR model for the jth SNP is $\alpha_{j}=\beta_{j}^{⊤}\theta +\gamma_{j}^{U}+\gamma_{j}^{C},\rho^{U}=Var\left(\gamma_{j}^{U}\right)/\mathrm{Var}\left(\beta_{j}^{⊤}\theta \right)$ and $\rho^{C}=\mathrm{Var}\left(\gamma_{j}^{C}\right)/Var\left(\beta_{j}^{⊤}\theta \right)$
**Step 1**. Using $n_{x},n_{Y},k_{xY},K_{xx}$, and $R_{xY}$, calculate the variance-covariance matrix of GWAS estimation errors ($\left.\Omega \right)$) using the methods in LeBlanc et al. (2018).
**Step 2**. Define the exposure genetic covariance matrix $\Sigma_{\beta \beta }={DR}_{\beta \beta }D$ where $D=diag\left(h_{1},\ldots ,h_{p}\right)$. Draw the (Mp)×1 vector of true effect sizes for causal exposure SNPs from $\mathrm{Normal}\left(0,M^{-1}\Sigma_{\beta \beta }\;⊗\;R\right)$ and reshape to be the M×p matrix B in which rows correspond to SNPs and columns to exposures. If R=I, we directly generate M true effect sizes from $\mathrm{Normal}\left(0,M^{-1}\Sigma_{\beta \beta }\right)$ to construct the matrix B.
**Step 3**. Draw vector of UHP effects $\left(\gamma^{U}\right)$ from $Normal\left(0,M^{-1}\rho^{U}\theta^{⊤}\Sigma_{\beta \beta }\theta \right)$ for M SNPs then fix all but the first $m^{U}$ elements to be 0.
**Step 4**. Draw CHP effects from $\gamma^{C}∼\mathrm{Normal}\left(0,M^{-1}\rho^{C}\theta^{⊤}\Sigma_{\beta \beta }\theta \right)+B\theta \left(-1+\zeta^{C}\right)$ then fix all but the next $m^{C}$ elements to be 0, which ensures that UHP and CHP SNPs do not overlap. The purpose of adding the factor $B\theta \left(-1+\zeta^{C}\right)$ is to make the correlation between Bθ and $\gamma^{C}$ negative by default, where the magnitude of this correlation is controlled by the user-specified value of $\zeta^{C}$. See Figure 4 for an example when $\zeta^{C}$ was −0.5.
**Step 5**. Calculate true associations between the M causal exposure SNPs and the outcome $\alpha =B\theta +\gamma^{U}+\gamma^{C}$.
**Step 6**. Draw GWAS estimation errors $\left(w_{\alpha },W_{\beta }\right)$ for (α,B) from Normal(0,I,Ω).
**Step 7**. Calculate the GWAS estimates $\hat{\alpha }=\alpha +w_{\alpha }$ and $\hat{B}=B+W_{\beta }$.
**Step 8**. For each jth SNP, draw estimated squared standard errors for ${\hat{\alpha }}_{j}$ from $n_{Y}\chi^{2}\left(n_{Y}-1\right)/\left(n_{Y}-1\right)$ and for ${\hat{\beta }}_{jk}$ for each of the kth exposures from $n_{Xk}\chi^{2}\left(n_{Xk}-1\right)/\left(n_{Xk}-1\right)$, where $\chi^{2}(a)$ is random chi-square distributed variate with a degrees of freedom.
**Step 9**. Apply LD and p-value pruning at the LD threshold $\kappa^{2}$ and p-value threshold τ, or reduce the set of SNPs to just IVs which achieve an average *F*-statistic of *F* across each exposure. The new set of SNPs which serve as IVs are placed into the set 𝒮.
**Step 10**. Standardize GWAS estimates according to user input
**Step 11**. Draw the matrix of estimated LD correlations between the IVs ${\hat{R}}_{𝒮}$ from $\text{Wishart}(n_{ref},R_{𝒮})$. If the user sets $n_{\text{ref}}$ to infinity, then this step is skipped and ${\hat{R}}_{𝒮}$ is equal to $R_{𝒮}$.
**Ensure**: The set of IVs 𝒮; their standardized GWAS estimates for the exposures ($\left({\hat{B}}_{𝒮}\right)$ and the outcome $\left({\hat{\alpha }}_{𝒮}\right)$ as the R objects bx and by; the corresponding GWAS-estimated standard errors $\hat{SE}\left({\hat{B}}_{𝒮}\right)$ and $\hat{SE}\left({\hat{\alpha }}_{𝒮}\right)$ as bxse and byse; indications for each IV if it was generated as a valid, UHP, or CHP SNP in IVType; true causal effects θ as theta; the matrix of correlations between GWAS estimation errors Ω as RhoME, with elements corresponding to the outcome in the first row and column; the matrices of true and estimated LD between the IVs $R_{𝒮}$ and ${\hat{R}}_{𝒮}$ as LDMatrix and LDhatMatrix.

## 3 | SIMULATION SETTINGS

**FIGURE 3:**
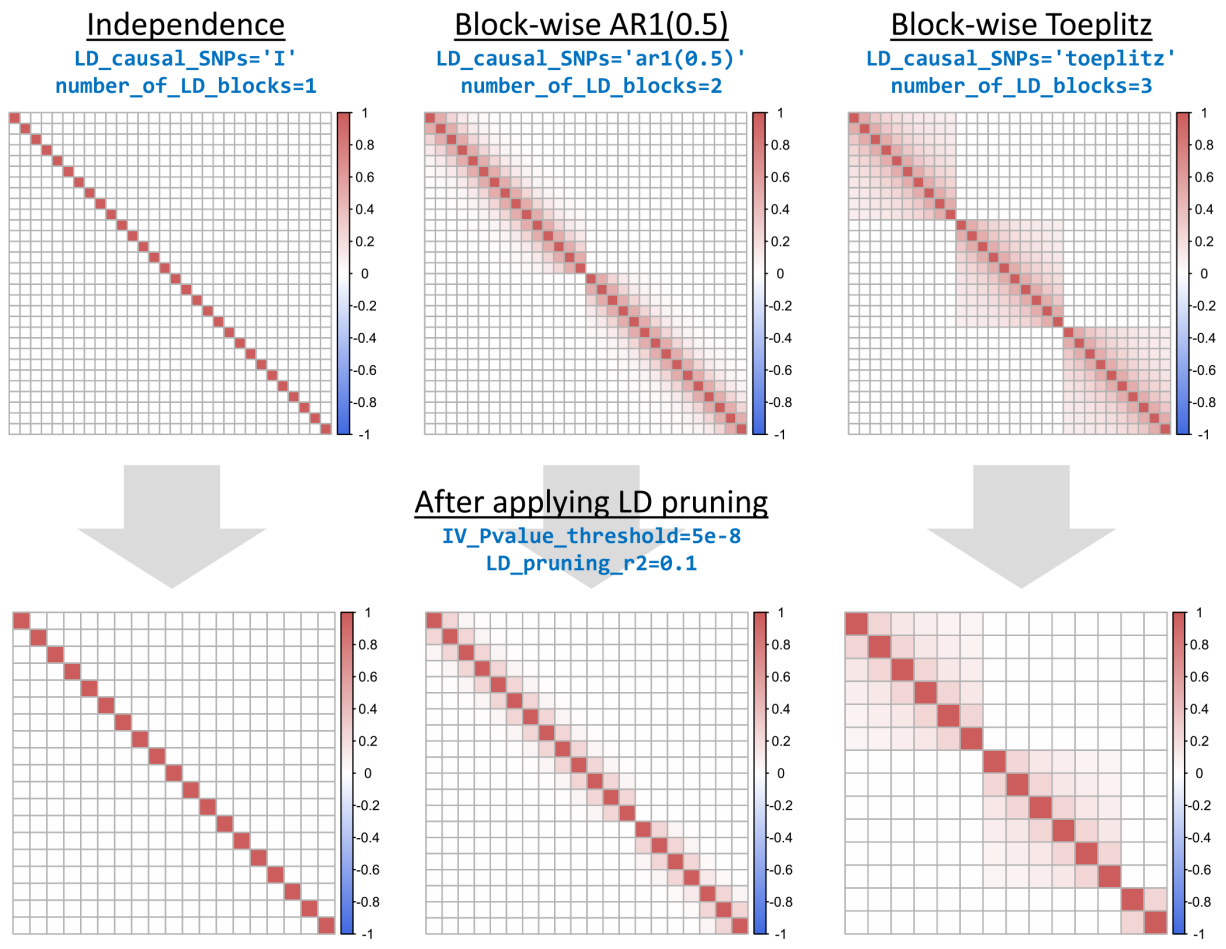
This figure provides examples of covariance structures which can be generated using simple expressions in simmrd. A first-order autoregressive covariance structure with correlation parameter a is generated by using “ar1(a),” a Toeplitz structure by using “toeplitz,” and an independence structure using “I.” Multiple parameters in simmrd receive the specification of a covariance structure as input, and a complete list of all parameters and their input is presented in the appendix for both the generate_individual() and generate_summary() functions.

In this section, we describe the conditions under which GWAS summary data can be generated and how some particular conditions are achieved. Users can generate GWAS summary data that spans a wide spectrum of scenarios defined by GWAS sample overlap, instrument strength, UHP, CHP, LD correlations between IVs, imprecise LD estimation, IV selection, and direct versus indirect causal effect sizes. To make the standardization of simulations performed by the broader community of MR researchers, we have also made a comprehensive set of default setups available for researchers to directly use (see Section 4 for more details). To achieve weak instruments, users can specify the exact *F*-statistic which their IV set achieves. In the case of multiple exposures, users specify the mean F-statistic which each exposure achieves in the MVMR IV set. Exact *F*-statistics are achieved by excluding SNPs with the largest effect sizes, thus also reducing the exposure heritability explained by the IVs. Correlated SNPs are generated by randomly sampling the true SNP-exposure(s) associations $B={\left(\beta_{j}\right)}_{j=1}^{M}$ from a matrix normal distribution with row-wise covariance that is the LD matrix R and column-wise covariance that is the matrix of genetic correlations between the exposures, $\Sigma_{\beta \beta }$. For any covariance matrix input, including the LD matrix, users can specify either independence, autoregressive, or Toeplitz structure using simple string expressions, some examples of which are presented in Figure 3.

We finally note here that MR methods which rely on genome-wide GWAS summary statistics (i.e., not just those for the selected IVs) for reasons other than to calculate $\mathrm{Cov}(\hat{B},\hat{\alpha })$ may not be able to solely use simmrd in their simulations, though it may still provide some support when, for example, creating block-diagonal LD matrices, fixing the *F*-statistic of the IV set, or pruning SNPs by LD and genetic association *p*-value. Such methods generally include Bayesian estimators which require genome-wide summary statistics to set and/or update parameterizations of prior distributions (see the appendix for a list). simmrd is technically capable of generating millions of SNP-phenotype association estimates for multiple traits without performing IV selection, but it will not generate any SNP-phenotype associations under null models in which the true association effect size is 0. MR methods which rely on genome-wide GWAS summary statistics only for the purposes of calculating $\mathrm{Cov}(\hat{B}-B,\hat{\alpha }-\alpha )$ can still use simmrd since our software will directly return the true $\mathrm{Cov}(\hat{B}-B,\hat{\alpha }-\alpha )$ using the methods of LeBlanc et al. (2018). These methods include Lorincz-Comi et al. (2023), Mounier and Kutalik (2023), Zhao, Wang, et al. (2020), Lin et al. (2023), and Cheng et al. (2020).

**FIGURE 4:**
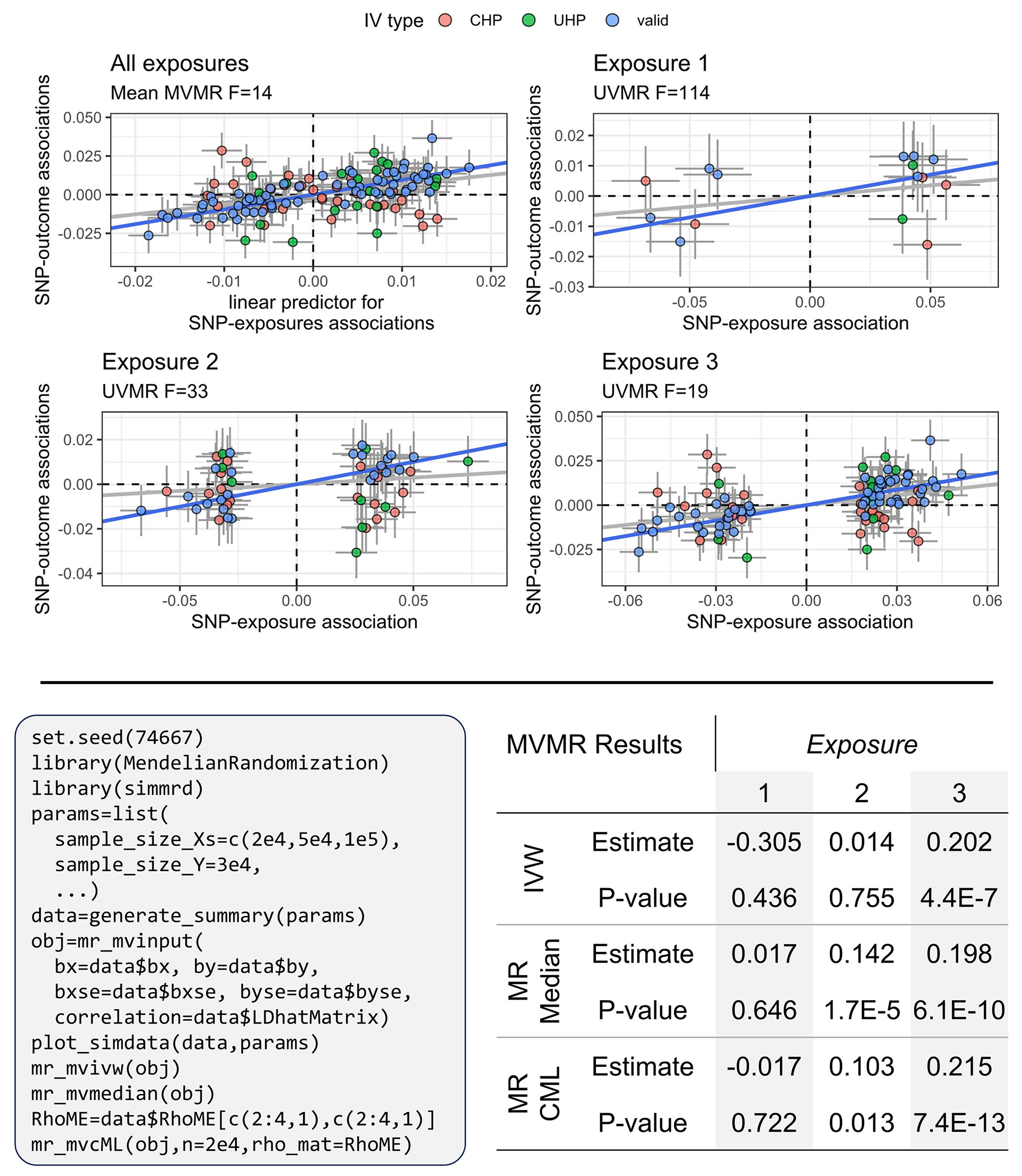
The top panel of this figure shows an example of the plots that plot_simdata(data,params) produces where params is a user-defined named list of input parameters and data is the stored output of either generate_individual (params) or generate_summary (params). Displayed on the *x*-axis of the top-left plot is the standardized linear predictor used in multivariable $MR(\overset{ˆ}{B}\overset{ˆ}{\theta })$ and the *y*-axis is the estimated association of the IVs with the outcome in standardized scale. The blue line is the IVW estimate using only the valid IVs (blue points), whereas the gray line is the IVW estimate using all IVs (blue, orange, and green points). All other plots are scatterplots of estimated SNP-exposure and SNP-outcome associations using only IVs that were selected separately for each exposure using the criteria specified by the user for MVMR. Displayed under the titles of each plot are *F*-statistics representing instrument strength (Burgess & Thompson, 2011). For the top-left plot which includes multiple exposures, the displayed *F*-statistic is the median F-statistic across all exposures using the full set of MVMR IVs. Determinations of the IV types (“valid,” “CHP,” or “UHP”) correspond to their status in the full MVMR IV set and not in the exposure-specific IV sets. The left figure in bottom panel contains R code to generate the figure in the top panel and numerical results in the table on the right, showing an example of how simmrd integrates with the popular MendelianRandomization R package (Yavorska & Burgess, 2017). The object params of simmrd input parameters is abbreviated to save space but is available in complete form in the example_params.R file on our Github repository. Note, the MR-CML (Lin et al., 2023) method assumes that the elements of the GWAS estimation error correlation matrix Ω corresponding to the outcome are in the bottom-right position, whereas simmrd automatically places them in the top-left position, hence why the object RhoME was created in the R code.

Figure 4 shows an example of data generated using generate_summary() for three exposures and how simmr integrates with the popular MendelianRandomization R package (Yavorska & Burgess, 2017). The top panel of this figure displays scatterplots which are the direct output of the plot_simdata() function included with the simmrd R package. For multiple exposures, plot_simdata() will return the both the bivariate SNP-exposure and SNP-outcome association estimates using exposure-specific IV sets and the association between the linear predictor $\hat{B}\hat{\theta }$ and $\overset{ˆ}{\alpha }$. Table 1 shows the time in seconds required to generate simulated MR data for a range of GWAS sample sizes and numbers of causal SNPs using both the generate_summary() and generate_individual() functions. These results indicate that the generate_summary() function can generate GWAS summary statistics and perform IV selection in one-tenth of a second or less for all scenarios. Alternatively, the generate_individual() function can take up to 2min to generate a single simulated data set for 1 million nonoverlapping exposure and outcome GWAS samples and 500 causal exposure SNPs. Still, many scenarios can be completed in less than 1 s, and simulation times increase more rapidly as GWAS sample sizes increase than as the number of causal SNPs increase.

## 4 | SOFTWARE

simmrd is an R package (R Core Team, 2021) and can be installed from our Github repository: https://github.com/noahlorinczcomi/simmrd. The simmrd software is open-source and intended to be iteratively improved by researchers to allow for greater functionality as new methodological developments are made. Changes to simmrd are tracked automatically, and earlier versions can be restored at any time. At our Github repository, we have provided the set of default simulation settings in the default_setups folder which researchers can use to perform simulations under pre-defined settings. These setups are stored in files containing parameters to give the generate_summary() function and cover five basic scenarios: (i) CHP, (ii) UHP, (iii) UHP + CHP, (iv) weak IVs, (v) UHP + CHP+weak IVs. Within each scenario, there are also multiple conditions of GWAS sample size (30 vs. 100K), GWAS sample overlap (0% vs. 100%), number of causal exposure SNPs (100 vs. 500), and number of exposures (1 vs. 3). A complete tutorial of how to use simmrd is also available on our Github repository.

## 5 | INPUT AND OUTPUT

Whether users of simmrd are generating GWAS summary data using individual-level data generate_individual() or just directly using generate_summary(), they are required to specify a named list of input parameters. The named items in this list are specific to each data-generating function, and we provide examples of how to create these lists in our tutorial provided at our Github repository. This is the only user input required for users to generate GWAS summary data to use in MR simulations. Here, we demonstrate how this list of parameters is used and state the output of both generate_summary() and generate_individual(). Let params be the R object which is the named list of parameters. After running either data=generate_individual (params) or data=generate_summary (params), the following summary data will be present in the named list data: (i) standardized SNP association estimates between all IVs and each exposure in the R matrix object bx, (ii) their corresponding standardized standard errors in bxse, (iii) standardized estimates of SNP association between each IV and the outcome in the vector by, (iv) their corresponding standardized standard errors in byse, (v) all corresponding unstandardized estimates of SNP association between the exposures and outcome and their corresponding standard errors (bx_unstd, bxse_unstd, by_unstd, byse_unstd), (vi) an indication for each IV if it was generated under a non-UHP/CHP, UHP, or CHP model in the vector IVType, (vii) the matrix of true LD correlations between the IVs (LDMatrix), (viii) the matrix of estimated LD correlations between the IVs (LDhatMatrix), (ix) the true causal effects of each exposure on the outcome in the vector theta, and (x) the matrix of correlations between GWAS estimation errors for the outcome and all exposures in the matrix RhoME. Users can view a summary visualization of their simulated data by executing plot_simdata(data,params) to view the scatterplots of SNP-exposure (s) and SNP-outcome association estimates.

## 6 | CONCLUSION

There is currently no standard simulation framework for performing simulations in Mendelian Randomization research. Different researchers have independently performed simulations designed to reflect similar real-world conditions, but the performance of the same methods can vary greatly. The MR literature is replete with MR estimators, with each at some point having a demonstrated advantage over others in simulation. The transferability of their performance to real world settings may be in question if the advantages can only be reached under specific conditions. We present simmrd, an open-source software for performing simulations to evaluate the performance of Mendelian Randomization methods. Researchers can directly modify the simmrd source code at our Github repository. It is our intention that the community will use this opportunity to establish an accepted procedure for performing simulations in MR. As simmrd is refined and expanded, we expect it will provide a useful tool to facilitate future MR method development and evaluation.

## Figures and Tables

**TABLE 1:** Displayed values are the times in seconds to generate a single set of simulated GWAS summary statistics for three exposures using either generate_summary() or generate_individual() as the GWAS sample size (N) and number of causal exposure SNPs changes.

simmrd run time (s) Causal exposure SNPs	*N* = 10 K	*N* = 30 K	*N* = 50 K	*N* = 100 K	*N* = 500 K	*N* = 1 M
generate_summary()						
10	<0.001	<0.001	<0.001	<0.001	<0.001	<0.001
50	0.001	0.001	0.001	0.001	0.001	0.002
100	0.002	0.001	0.002	0.001	0.002	0.001
250	0.003	0.002	0.003	0.003	0.004	0.004
500	0.004	0.004	0.006	0.007	0.010	0.017

generate_individual()						
10	0.023	0.054	0.082	0.190	9.621	4.609
50	0.090	0.254	0.424	0.850	4.445	8.807
100	0.173	0.500	0.845	3.501	9.066	19.997
250	0.391	1.154	2.073	6.127	25.685	51.084
500	0.881	4.455	4.191	8.598	53.099	120.809

*Note*: The causal SNPs were generated without LD structure and the exposure and outcome GWAS samples did not overlap. Using generate_individual(), the exposure GWASs overlapped completely.

## APPENDIX A

### A.1. Current MR methods

We mentioned in Section 1 that at least 45 statistical methods for performing Mendelian randomization (MR) exist. Over 40 current MR methods can directly use the simmrd software without any additional simulation data and are IVW (Burgess et al., 2013), dIVW (Ye et al., 2021), pIVW (Xu et al., 2022), MR-Egger (Bowden et al., 2016), MRAID (Yuan et al., 2022), MRMix (Qi & Chatterjee, 2019), MR-cML (Xue et al., 2021), MVMR-cML (Lin et al., 2023), MR-PRESSO (Verbanck et al., 2018), IMRP (Zhu et al., 2021), MR-Median (Bowden et al., 2016), MR-MaxLike (Burgess, Dudbridge, et al., 2016), MR-Corr2 (Cheng, Qiu, et al., 2022), MR-Robust (Rees et al., 2019), MR-Lasso (Kang et al., 2016), MR-Conmix (Burgess et al., 2020), MR-CUE, EMIC (Jiang, Miao, et al., 2022), MR-Mode (Hartwig et al., 2017), MRBEE (Lorincz-Comi et al., 2023), MR-Lap (Mounier & Kutalik, 2023), the Wald test (Palmer et al., 2008), MR-BMA (Zuber et al., 2020), MR-Robin (Gleason et al., 2020), JAM (Newcombe et al., 2016), MR using factor analysis (Patel et al., 2023), moPMR-Egger (Liu et al., 2021), MR-Clust (Foley et al., 2021), MR using PCA (Burgess et al., 2017), MR-CUE (Cheng, Zhang, et al., 2022), mixIE (Lin et al., 2021), MRMO (Deng et al., 2022), BWMR (Zhao, Ming, et al., 2020), sisVIVE (Kang et al., 2016), MR-LDP (Cheng et al., 2020), MR-CIP (Xu et al., 2021), MR-PATH (Iong et al., 2020), BMRE (Schmidt & Dudbridge, 2018), MR-link (van Der Graaf et al., 2020), CoJo (Yang et al., 2012), and GRAPPLE (Wang, Zhao, et al., 2021). MR methods which require genome-wide GWAS summary statistics that is, not just those for the candidate IV set, include CAUSE (Morrison et al., 2020), MR-Horse (Grant & Burgess, 2023), BayesMR (Bucur et al., 2020), and OMR (Wang, Gao, et al., 2021), and those which require individual-level data include BMRMO (Deng et al., 2023).

### A.2. Using generate_individual() and generate_summary()

To generate data using simmrd, two steps must be completed. First, users create a named list. Each element of this list is a parameter value and the name of each element is the parameter name. generate_individual() and generate_summary() use different lists of parameters, as Figure 2 demonstrates. Assume for example, that the user has one list of parameters assigned to the R object individual_params which they intend to use with generate_individual() and another named summary_params which they intend to use with generate_summary(). The first few elements of summary_params or individual_params may look like:
list(sample_size_Xs=5e4, sample_size_Y=3e4, number_of_exposures=2,).

Simulated data can be generated by running either the
data=generate_individual (individual_params)

or
data=generate_summary (summary_params)
commands in R. The returned object stored as data is a named list of elements which are GWAS summary data. We provide a description of each parameter for generate_individual() and generate_summary() below and complete examples of how to use simmrd at our Github repository: https://github.com/noahlorinczcomi/simmrd.

The parameters that generate_individual() uses are the following:

1. [sample_size_Xs]: sample sizes of the p exposure GWASs; either a scalar applied to all exposures or a p-length vector of scalars applied to each exposure element-wise.
2. [sample_size_Y]: sample size of the outcome GWAS; scalar.
3. [prop_gwas_overlap_Xs_and_Y]: proportions of sample overlap between exposure and outcome GWAS; either a scalar applied to all exposure-outcome pairs or a p-length vector of scalars applied to each exposure-outcome pair element-wise.
4. [number_of_exposures]: number of exposures; scalar.
5. [phenotypic_correlation_Xs]: phenotypic correlations between the exposures; scalar, actual matrix of correlations, or string value which is one of “I” for independent exposures, “toeplitz” for a correlation matrix with Toeplitz structure, or (e.g.) “ar1(0.25)” for a correlation matrix with first-order autoregressive structure with correlation parameter 0.25.
6. [genetic_correlation_Xs]: genotypic correlations between the exposures; scalar, actual matrix of correlations, or string value which is one of “I” for independent exposures, “toeplitz” for a correlation matrix with Toeplitz structure, or (e.g.) “ar1(0.25)” for a correlation matrix with first-order autoregressive structure with correlation parameter 0.25.
7. [Xs_variance_explained_by_U]: variance in the exposure(s) explained by the confounder conditional on the causal exposure SNPs (the total variance of the exposure(s) is/are 1); scalar applied to all exposures or p-length vector applied to each exposure element-wise.
8. [Y_variance_explained_by_Xs]: variance in the outcome explained by the exposure(s) conditional on the confounder and direct effects of causal exposure SNPs (the variance of the outcome is 1, making the value(s) provided here directly proportional to the causal effect sizes); scalar applied to all exposures or p-length vector applied to each exposure element-wise.
9. [signs_of_causal_effects]: signs of the causal effects; scalar applied to all exposures or p-length vector of 1s, −1s, or 0s applied to each exposure element-wise.
10. [Y_variance_explained_by_U]: variance in the outcome explained by the confounder conditional on the exposure(s) and direct effects of the causal exposure SNPs (the total variance of the outcome is 1); scalar.
11. [number_of_causal_SNPs]: number of causal exposure SNPs; scalar.
12. [Xs_variance_explained_by_g]: variance in the exposure(s) explained by the causal SNPs (the total variance of each exposure is 1); scalar applied to each exposure or p-length vector applied to each exposure element-wise.
13. [number_of_UHP_causal_SNPs]: number of causal exposure SNPs which have UHP effects on the outcome; scalar.
14. [number_of_CHP_causal_SNPs]: number of causal exposure SNPs which have CHP effects on the outcome; scalar.
15. [Y_variance_explained_by_UHP]: variance in the outcome explained by direct effects of the causal exposure SNPs conditional on the exposure(s) and confounder (the total variance of the outcome is 1); scalar.
16. [U_variance_explained_by_CHP]: variance in the confounder explained by the causal exposure SNPs (the total variance of the confounder is 1).
17. [LD_causal_SNPs]: LD correlation structure of the causal SNPs; matrix of LD correlations between all causal SNPs (dimensions must match the value given to number_of_causal_SNPs), or string value which is one of “I” for independent SNPs, “toeplitz” for an LD correlation matrix with Toeplitz structure, or (e.g.) “ar1(0.25)” for an LD correlation matrix with first-order autoregressive structure with correlation parameter 0.25.
18. [number_of_LD_blocks]: number of independent LD blocks in the LD correlation matrix; scalar.
19. [MR_standardization]: type of standardization to apply to GWAS summary statistics; one of “none” for no standardization or “Z” for Z-score standardization (i.e., dividing GWAS association estimates by their estimated standard error). Note, minor allele frequency is not considered because genotypes in all simulations are assumed to have mean 0 and variance 1.
20. [simtype]: one of “winners” or “weak” for a winner’s curse, which forces IV selection using the *p*-value threshold IV_Pvalue_threshold, or weak instruments simulation, which fixes the mean F-statistic across exposures at fix_Fstatistic_at and does not perform SNP selection using *p*-values; string.
21. [fix_Fstatistic_at]: the *F*-statistic value to achieve for all exposures on average using the full MVMR IV set (the UVMR IV set if only one exposure is considered); scalar.
22. [MVMR_IV_selection_type]: type of method used to calculate *p*-values to use in selecting IVs based on *p*-value; one of “union,” in which MVMR IVs are the union set of exposure-specific IV sets, or “joint” in which each IV has a *P*-value less than “IV_Pvalue_threshold” in a p-degree of freedom joint chi-square test of association between the SNP and exposures.
23. [IV_Pvalue_threshold]: only SNPs with a *p*-value (see “MVMR_IV_selection_type” to see where this *p*-value comes from) less than this threshold will be considered as candidate IVs; scalar.
24. [LD_pruning_r2]: for two pairs of SNPs that have a squared LD correlation greater than this threshold, only the one with the smaller *p*-value will be considered as a candidate IV; scalar.
25. [N_of_LD_ref]: sample size of the LD reference panel; scalar or “Inf.”

The parameters that generate_summary() uses are the following:

1. [sample_size_Xs]: sample sizes of the p exposure GWASs; either a scalar applied to all exposures or a p-length vector of scalars applied to each exposure element-wise.
2. [sample_size_Y]: sample size of the outcome GWAS; scalar.
3. [prop_gwas_overlap_Xs_and_Y]: proportions of sample overlap between exposure and outcome GWAS; either a scalar applied to all exposure-outcome pairs or a p-length vector of scalars applied to each exposure-outcome pair element-wise.
4. [number_of_exposures]: number of exposures; scalar.
5. [number_of_causal_SNPs]: number of causal exposure SNPs; scalar.
6. [number_of_UHP_causal_SNPs]: number of causal exposure SNPs which have UHP effects on the outcome; scalar.
7. [number_of_CHP_causal_SNPs]: number of causal exposure SNPs which have CHP effects on the outcome; scalar.
8. [ratio_of_UHP_variance]: ratio of variance of UHP causal effects to the linear predictor of vertical horizontal pleiotropy effects; scalar.
9. [ratio_of_CHP_variance]: ratio of variance of CHP causal effects to the linear predictor of vertical horizontal pleiotropy effects; scalar.
10. [CHP_correlation]: *ζ^C^* in Step 4 of Algorithm 2, which controls the magnitude of correlation between CHP and vertical horizontal pleiotropy IV effects; scalar.
11. [simtype]: one of “winners” or “weak” for a winner’s curse, which forces IV selection using the *p*-value threshold IV_Pvalue_threshold, or weak instruments simulation, which fixes the mean F-statistic across exposures at fix_Fstatistic_at and does not perform SNP selection using *p*-values; string.
12. [fix_Fstatistic_at]: the *F*-statistic value to achieve for all exposures on average using the full MVMR IV set (the UVMR IV set if only one exposure is considered); scalar.
13. [prop_gwas_overlap_Xs]: proportion of overlap between exposure GWAS samples; scalar applied to all exposure GWAS, *p* × *p* symmetric matrix of actual overlap proportions, or string value which is one of “I” for no overlap, “toeplitz” for a proportion overlap matrix with Toeplitz structure, or (e.g.) “ar1(0.25)” for a proportion overlap matrix with first order autoregressive structure with correlation parameter 0.25.
14. [phenotypic_correlation_Xs]: phenotypic correlations between the exposures; scalar, actual matrix of correlations, or string value which is one of “I” for independent exposures, “toeplitz” for a correlation matrix with Toeplitz structure, or (e.g.) ”ar1(0.25)” for a correlation matrix with first order autoregressive structure with correlation parameter 0.25.
15. [genetic_correlation_Xs]: genotypic correlations between the exposures; scalar, actual matrix of correlations, or string value which is one of “I” for independent exposures, “toeplitz” for a correlation matrix with Toeplitz structure, or (e.g.) “ar1(0.25)” for a correlation matrix with first order autoregressive structure with correlation parameter 0.25.
16. [phenotypic_correlations_Xs_and_Y]: phenotypic correlations between the exposures and outcome; scalar applied to each exposure or a p-length vector of values applied to each exposure element-wise.
17. [true_causal_effects]: true causal effects of the exposure(s) on the outcome; scalar applied to each exposure or p-length vector applied to each exposure element-wise.
18. [Xs_variance_explained_by_g]: variance in the exposure(s) explained by the causal SNPs; scalar applied to each exposure or p-length vector applied to each exposure element-wise.
19. [LD_causal_SNPs]: LD correlation structure of the causal SNPs; matrix of LD correlations between all causal SNPs (dimensions must match the value given to number_of_causal_SNPs), or string value which is one of “I” for independent SNPs, “toeplitz” for an LD correlation matrix with Toeplitz structure, or (e.g.) “ar1(0.25)” for an LD correlation matrix with first order autoregressive structure with correlation parameter 0.25.
20. [number_of_LD_blocks]: number of independent LD blocks in the LD correlation matrix; scalar.
21. [MR_standardization]: type of standardization to apply to GWAS summary statistics; one of “none” for no standardization or “Z” for *Z*-score standardization (i.e., dividing GWAS association estimates by their estimated standard error). Note, minor allele frequency is not considered because genotypes in all simulations are assumed to have mean 0 and variance 1.
22. [MVMR_IV_selection_type]: type of method used to calculate *p*-values to use in selecting IVs based on *p*-value; one of “*union*,” in which MVMR IVs are the union set of exposure-specific IV sets, or “joint,” in which each IV has a P-value less than “IV_Pvalue_threshold” in a p-degree of freedom joint chi-square test of association between the SNP and exposures.
23. [IV_Pvalue_threshold]: only SNPs with a *p*-value (see “MVMR_IV_selection_type” to see where this *p*-value comes from) less than this threshold will be considered as candidate IVs; scalar.
24. [LD_pruning_r2]: for two pairs of SNPs that have a squared LD correlation greater than this threshold, only the one with the smaller *p*-value will be considered as a candidate IV; scalar.
25. [N_of_LD_ref]: sample size of the LD reference panel; scalar or “Inf.”

### Availability and requirements

Project name: simmrd

Project home page: https://github.com/noahlorinczcomi/simmrd

Operating system(s): Platform independent

Programming language: R

Other requirements: R 4.1.1 or higher; mvnfast (required) and ggplot2 (optional) R packages

License: MIT

## Abbreviations:

DAG: directed acyclic graph
GWAS: Genome-Wide Association Study
LD: linkage disequilibrium
MR: Mendelian randomization
MVMR: multivariable Mendelian randomization
SNP: single-nucleotide polymorphism
UVMR: univariable Mendelian randomization
